# Study protocol for The GOAL Trial: comprehensive geriatric assessment for frail older people with chronic kidney disease to increase attainment of patient-identified goals—a cluster randomised controlled trial

**DOI:** 10.1186/s13063-023-07363-4

**Published:** 2023-05-30

**Authors:** B Logan, AK Viecelli, DW Johnson, EM Aquino, J Bailey, TA Comans, LC Gray, CM Hawley, LE Hickey, M Janda, A Jaure, MD Jose, E Kalaw, C Kiriwandeniya, M Matsuyama, G Mihala, KH Nguyen, E Pascoe, JD Pole, KR Polkinghorne, D Pond, R Raj, DM Reidlinger, N Scholes-Robertson, J Varghese, G Wong, RE Hubbard, on behalf of The GOAL Trial Investigators, Graham Buckle, Phil Carswell, Joanne Cerni, Michael G. Collins, Amanda Elms, John Fanning, Karen Fischer, Adam Flavell, Emily H. Gordon, Natalie Grainer, Stella Green, Chandana Guha, Samantha Hand, Moira Hibbs, Bronwyn Hockley, Rachael Irvine, Ibrahim Ismail, Shilpanjali Jesudason, George Kan, Debbie Kennedy, Vinod Khelgi, Shannon Kokoszka, Anoushka Krishnan, Janelle Leadbitter, Diana Leary, Angela Makris, Khalilah Katherine Marquez, David McIntyre, Penelope Murie, Karina Murphy, Nancye Peel, Xiaodan Qiu, Madeleine Rapisardi, Matthew A. Roberts, Simon D. Roger, Mona Saade, Shaundeep Sen, Gerald Tataro, Brioney Weaver, Paul A. Yates, Belinda Yip

**Affiliations:** 1 Centre for Health Services Research, University of Queensland, Brisbane, Australia; 2 Australasian Kidney Trials Network, University of Queensland, Brisbane, Australia; 3 Department of Kidney and Transplant Services, Princess Alexandra Hospital, Brisbane, Australia; 4 Centre for Kidney Disease Research, Translational Research Institute, Brisbane, Australia; 5 Sydney School of Public Health, The University of Sydney, Sydney, Australia; 6 Renal Unit, Royal Hobart Hospital, Hobart, Australia; 7 School of Medicine, University of Tasmania, Hobart, Australia; 8 Global Brain Health Institute, Trinity College, Dublin, Ireland; 9 Dalla Lana School of Public Health, The University of Toronto, Toronto, Canada; 10 School of Public Health and Preventive Medicine, Monash University, Melbourne, Australia; 11 Department of Medicine, Monash University, Melbourne, Australia; 12 Department of Nephrology, Monash Health, Melbourne, Australia; 13 School of Rural Medicine, University of New England, Armidale, Australia; 14 Wicking Centre, University of Tasmania, Hobart, Australia; 15 School of Medicine, Western Sydney University, Sydney, Australia; 16 Department of Nephrology, Launceston General Hospital, Launceston, Australia; 17 Rural and Remote Health, College of Medicine and Public Health, Flinders University, Adelaide, Australia; 18 Centre for Kidney Research, The Children’s Hospital at Westmead, Sydney, Australia; 19 Centre for Transplant and Renal Research, Westmead Hospital, Sydney, Australia; 20 Department of Geriatric Medicine, Princess Alexandra Hospital, Brisbane, Australia

**Keywords:** Activities of daily living, Aged, Assessment, Chronic kidney disease, Cost-effectiveness, Dialysis, Cluster randomised controlled trial, Comprehensive geriatric assessment, Frail elderly, Frailty, Functional improvement, Goal attainment scaling, Goals, Goal setting, Process analysis, Quality of life, Reablement, Recovery of function, Treatment outcome

## Abstract

**Background:**

An increasing number of older people are living with chronic kidney disease (CKD). Many have complex healthcare needs and are at risk of deteriorating health and functional status, which can adversely affect their quality of life. Comprehensive geriatric assessment (CGA) is an effective intervention to improve survival and independence of older people, but its clinical utility and cost-effectiveness in frail older people living with CKD is unknown.

**Methods:**

The GOAL Trial is a pragmatic, multi-centre, open-label, superiority, cluster randomised controlled trial developed by consumers, clinicians, and researchers. It has a two-arm design, CGA compared with standard care, with 1:1 allocation of a total of 16 clusters. Within each cluster, study participants ≥ 65 years of age (or ≥ 55 years if Aboriginal or Torres Strait Islander (First Nations Australians)) with CKD stage 3–5/5D who are frail, measured by a Frailty Index (FI) of > 0.25, are recruited. Participants in intervention clusters receive a CGA by a geriatrician to identify medical, social, and functional needs, optimise medication prescribing, and arrange multidisciplinary referral if required. Those in standard care clusters receive usual care. The primary outcome is attainment of self-identified goals assessed by standardised Goal Attainment Scaling (GAS) at 3 months. Secondary outcomes include GAS at 6 and 12 months, quality of life (EQ-5D-5L), frailty (Frailty Index – Short Form), transfer to residential aged care facilities, cost-effectiveness, and safety (cause-specific hospitalisations, mortality). A process evaluation will be conducted in parallel with the trial including whether the intervention was delivered as intended, any issue or local barriers to intervention delivery, and perceptions of the intervention by participants. The trial has 90% power to detect a clinically meaningful mean difference in GAS of 10 units.

**Discussion:**

This trial addresses patient-prioritised outcomes. It will be conducted, disseminated and implemented by clinicians and researchers in partnership with consumers. If CGA is found to have clinical and cost-effectiveness for frail older people with CKD, the intervention framework could be embedded into routine clinical practice. The implementation of the trial’s findings will be supported by presentations at conferences and forums with clinicians and consumers at specifically convened workshops, to enable rapid adoption into practice and policy for both nephrology and geriatric disciplines. It has potential to materially advance patient-centred care and improve clinical and patient-reported outcomes (including quality of life) for frail older people living with CKD.

**Trial registration:**

ClinicalTrials.gov NCT04538157. Registered on 3 September 2020.

**Supplementary Information:**

The online version contains supplementary material available at 10.1186/s13063-023-07363-4.

## Administrative information

Note: the numbers in curly brackets in this protocol refer to a Standard Protocol Items: Recommendations for Interventional Trials (SPIRIT) checklist item numbers [1, 2]. The order of the items has been modified to group similar items (see http://www.equator-network.org/reporting-guidelines/spirit-2013-statement-defining-standard-protocol-items-for-clinical-trials/).

**Table:** 

Title {1}	Study protocol for The GOAL Trial: comprehensive geriatric assessment for frail older people with chronic kidney disease to increase attainment of patient-identified goals—a cluster randomised controlled trial
Trial registration {2a and 2b}	ClinicalTrials.gov: NCT04538157. Registered on: 3 September 2020
Protocol version {3}	Version 2.5, 22 March 2023
Funding {4}	National Health and Medical Research Council Targeted Research Grant: Research into Frailty in Hospital Care (APP1178519)
Author details {5a}	1. Centre for Health Services Research, University of Queensland, Brisbane, Australia. 2. Australasian Kidney Trials Network, University of Queensland, Brisbane, Australia. 3. Department of Kidney and Transplant Services, Princess Alexandra Hospital, Brisbane, Australia. 4. Centre for Kidney Disease Research, Translational Research Institute, Brisbane, Australia. 5. Sydney School of Public Health, The University of Sydney, Sydney, Australia. 6. Renal Unit, Royal Hobart Hospital, Hobart, Australia. 7. School of Medicine, University of Tasmania, Hobart, Australia. 8. Global Brain Health Institute, Trinity College, Dublin, Ireland. 9. Dalla Lana School of Public Health, The University of Toronto, Toronto, Canada. 10. School of Public Health and Preventive Medicine, Monash University, Melbourne, Australia. 11. Department of Medicine, Monash University, Melbourne, Australia. 12. Department of Nephrology, Monash Health, Melbourne, Australia. 13. School of Rural Medicine, University of New England, Armidale, Australia. 14. Wicking Centre, University of Tasmania, Hobart, Australia. 15. School of Medicine, Western Sydney University, Sydney, Australia. 16. Department of Nephrology, Launceston General Hospital, Launceston, Australia. 17. Rural and Remote Health, College of Medicine and Public Health, Flinders University, Adelaide, Australia. 18. Centre for Kidney Research, The Children's Hospital at Westmead, Sydney, Australia. 19. Centre for Transplant and Renal Research, Westmead Hospital, Sydney, Australia. 20. Department of Geriatric Medicine, Princess Alexandra Hospital, Brisbane, Australia.
Name and contact information for the trial sponsor {5b}	The University of Queensland acting through the Australasian Kidney Trials Network (AKTN) Email: aktn@uq.edu.au
Role of sponsor {5c}	The sponsor is the coordinating centre for the trial and is involved in overall study activities including study design, collection, management, analysis and interpretation of data, writing of the report, and decision to submit the report for publication.

## Introduction

### Background and rationale {6a}

Chronic kidney disease (CKD) is a non-communicable disease in which the structure and/or function of the kidneys have been irreversibly altered by one or more heterogeneous disease pathways, most commonly hypertension and diabetes, for a period of at least three months [3, 4]. Globally, it is recognised as a major contributor to morbidity and mortality, with an estimated population prevalence of 9.1% [5]. In Australia, CKD affects at least 1 in 10 adults (1.7 million people), is related to 16% of hospitalisations, and contributes to 10% of deaths [6, 7]. The prevalence of CKD increases exponentially with older age due to the increase in comorbid chronic diseases such as type 2 diabetes mellitus and hypertension, and an increased susceptibility to nephrotoxic agents and drugs [8]. Ninety per cent of people with moderate to severe CKD are aged 65 and above [7].

Older people with CKD have complex health needs. When compared to people seen by other specialists, those seen by kidney specialists have a higher number of comorbid conditions and medications, and more frequent institutionalisation [9]. This can make care planning challenging for older people, caregivers and the healthcare team. Managing older people with CKD will remain a pressing issue for individuals and the wider health system given the projected ageing of the population [10]. Through an enhanced understanding of this population, care planning can be more appropriate and better reflect their needs.

For those living with CKD, frailty is highly prevalent [11, 12]. Frailty is defined as a state of increased vulnerability to poor resolution of homeostasis after a stressor event, which increases the risk of adverse outcomes [13]. Frail older people are at higher risk for mortality, incident falls, hospitalisation, and worsening mobility and functional dependence [14–19]. In community-dwellers, frailty is associated with more frequent doctor visits, greater institutionalisation, social isolation, and poorer self-reported perceived health [16, 17]. Identification and quantification of frailty is important as it enables health professionals to personalise management to achieve patient-centred outcomes.

How best to manage and slow the progression of frailty is of interest to researchers and policy makers [20]. The British Geriatrics Society, Age UK, and the Royal College of General Practitioners have issued Best Practice Guidelines which recommend a comprehensive geriatric assessment (CGA) be completed for all older people identified as being frail [21]. CGA seeks to improve person-centred care by first identifying their medical, functional and psychosocial problems, and then tailoring coordinated management plans to address them [22–24]. It is of proven benefit for older people in hospital, increasing the likelihood that they will be alive and residing in their own homes at follow-up [22]. It can also prevent a decline in function and improve the quality of life for frail older community dwellers [25]. Despite CGA being featured in practice guidelines, its evidence for reducing frailty in community dwellers is of low certainty [26, 27], and the details of the essential elements of a CGA differs among clinicians [28].

To date, no research has been undertaken to specifically understand the efficacy and safety of CGA in frail older people with CKD. The GOAL trial will be the first study of the efficacy, safety and cost-effectiveness of CGA in this population, internationally. It will do so with an appreciation of the importance of person-centred care and the role patient-identified goals have in underpinning it, given attainment of their own goals is the primary outcome. The trial’s clinical relevance and utility will also be enhanced through consumer involvement, via the Consumer Advisory Board, during the trial development, conduct and dissemination of results. It features an embedded process evaluation, which will contribute to better understanding of the essential components of CGA delivered and their importance for outcome achievement.

### Objectives {7}

The primary hypothesis is that frail older people with stages 3–5/5D chronic kidney disease (CKD) who receive CGA, as opposed to usual care, have greater attainment of their self-identified goals.

The secondary hypotheses are that CGA being administered to frail older people with stages 3–5/5D CKD improves the quality of life, reduces frailty and mortality, lessens hospital admission rates and length of stay, and decreases Residential Aged Care Facility (RACF) admissions, whilst being safe and cost-effective. A process evaluation will enable the establishment of features of CGA delivery that increase the likelihood that the intervention will be effective.

### Trial design {8}

The GOAL Trial is a pragmatic, multicentre, superiority, open-label cluster randomised controlled trial. While the intervention will be implemented at the participant level, randomisation at the cluster (hospital) level has been chosen to avoid contamination of the intervention. The Australasian Kidney Trials Network (AKTN, The University of Queensland) is the sponsor and coordinating centre for the trial.

## Methods: participants, interventions, and outcomes

### Study setting {9}

Recruitment will occur at the nephrology outpatient clinics (the clusters) of 16 public hospitals across Australia, with a mix of tertiary and peripheral sites. Each hospital will form a cluster. For a cluster to be eligible for inclusion, it is expected they have a minimum of 55 older people who meet the trial’s participant eligibility criteria (noted below). The list of study sites is available elsewhere (see Supplementary file 1).

### Eligibility criteria {10}

#### Inclusion criteria

For inclusion in screening, an individual must meet both of the following criteria:

- Have moderate to severe CKD as determined by the treating nephrologist
  - ◦ Stage 3 = eGFR 30–59 mL/min/1.73 m.^2^
  - ◦ Stage 4 = eGFR 15–29 mL/min/1.73 m.^2^
  - ◦ Stage 5/5D = eGFR below 15 mL/min/1.73m^2^, including those receiving dialysis
- Be aged ≥ 65 years, or ≥ 55 if Aboriginal and/or Torres Strait Islander (First Nation’s Australians)

For those who progress to enrolment, inclusion as a study participant requires a Frailty Index (FI) of > 0.25 as measured by the FI Short Form, a validated measure in the nephrology outpatient setting [29].

An FI cut-off at 0.25 has been selected (robust [FI ≤ 0.25] or frail [FI > 0.25]) as research has demonstrated individuals who were clinically ‘apparently vulnerable’ had a mean FI of 0.22, compared to those who were ‘mildly frail’ with mean FI of 0.27 [30]. Whilst the FI is not intended to be dichotomous, for the purposes of a study design that requires a value for participant recruitment, a cut-off at 0.25 is recognised as appropriate [31]. This approach is consistent with other work undertaken within the field of frailty research [32–36].

#### Exclusion criteria

The exclusion criteria are either:

- Estimated life expectancy (12 months), or
- Unable to provide informed consent and/or participate in the Goal Attainment Scaling (GAS) process due to cognitive impairment or another reason.

### Who will take informed consent? {26a}

During clinic appointments, the sites’ principal investigators or a delegate, will approach prospective trial participants to introduce the trial, describe the study, and answer questions. Prospective participants will be provided with the Patient Information Sheet and Consent Form (see Supplementary file 2). After discussing the trial, ample time will be given to the prospective participant to enquire about the trial and decide whether to participate. If informed consent is provided, a consent form will be signed. All those who consent to participate will have the FI Short Form tool administered by a research nurse or delegate. If the FI score is > 0.25, the individual will be enrolled in the study and proceed to the completion of baseline assessments.

Since the coronavirus disease of 2019 (COVID-19) has necessitated some telephone clinic appointments rather than face-to-face appointments, the consent process may be conducted in person or remotely. Verbal consent will be available prior to conducting screening assessments and when it is permitted according to local regulations. The site investigator, research nurse or delegate will obtain informed verbal consent prior to conducting the Frailty Assessment. All staff involved in obtaining consent and enrolling participants have received specific training in the trial and the requirements of Good Clinical Practice.

### Additional consent provisions for collection and use of participant data and biological specimens {26b}

The consent process includes a provision for data linkage of research observations with state government health information systems data. No additional biological samples outside those collected as part of routine clinical care are being collected from participants. There are no ancillary studies planned.

## Intervention

### Explanation for the choice of comparators {6b}

Participating sites assigned to the control group will provide participants with usual care as per each hospital’s standard operating procedures. This group will be compared to those participants at intervention group clusters who receive CGA as an intervention in addition to usual care.

At control sites, a letter will be issued to the treating General Practitioner (GP) to inform them of a participant’s enrolment in the trial.

### Intervention description {11a}

The intervention, CGA, is a consultation completed by a geriatrician. Geriatricians are specialists who routinely undertake this task in their role of managing patients aged over 65 years. It forms a key part of their specialist training. Overviewed in Table 1 are the various components outlined in the consensus guidelines, which may be included in CGA at the geriatrician’s discretion, as determined by the participant’s individual needs [23, 37].

**Table 1:** Domains of a comprehensive geriatric assessment—adapted from Welsh TJ, Gordon AL, Gladman JR. Comprehensive geriatric assessment—a guide for the non-specialist [23]; and, Parker SG, McCue P, Phelps K, McCleod A, Arora S, Nockels K, et al. What is Comprehensive Geriatric Assessment (CGA)? An umbrella review [37]

Domain	Components
Physical health	Comorbid conditions and disease severity Medication review Nutritional status
Psychological health	Cognition Mood and anxiety
Functioning	Mobility, balance and falls Personal and instrumental activities of daily living Transport
Social circumstances	Social networks (including family and informal support available) Accommodation
Future planning	Advance care planning Patient goals and priorities

For this trial, CGA will be delivered by the geriatrician in an outpatient clinic setting within 14 days of the baseline GAS. It is expected to take one hour (Medicare, Australia’s universal health insurance scheme, allocates 60 min for these assessments). The geriatrician will have access to the patient’s list of current medications and their medical history. When appropriate, the participant may also be accompanied by a support person (such as a spouse) who is allowed to provide collateral history to the geriatrician if warranted. This reflects routine practice.

At some hospitals, particularly teaching hospitals within the public health system, CGAs are conducted by Advanced Trainees in Geriatric Medicine with supervision from a consultant geriatrician. Advanced Trainees are doctors completing their final years of specialisation training. To increase the feasibility and generalisability of The GOAL Trial intervention, Advanced Trainees in Geriatric Medicine may complete the CGA provided they are supervised and the reports they generate are reviewed by the responsible geriatrician.

GPs often have insights into patients’ functional and cognitive problems that are not recognised or documented by hospital-based teams. The patient’s GP will be asked to consider making a referral to the geriatric outpatient clinic as per routine practice and asked to provide any additional relevant information that they feel would be relevant. This will occur once a participant is enrolled, and before they are seen in the outpatient clinic by the Geriatrician.

Following the CGA, the geriatrician will generate a letter to the treating nephrologist, GP and any other relevant specialists, which will include a comprehensive problem list and recommendations regarding an individualised care plan. The necessary referrals to allied health professionals and other specialists will be undertaken by the treating geriatrician where the site has their own Day Hospital or relevant outpatient service. Where such services are not available, the letter will request the assistance of the participant’s GP to make the required referrals to community-based providers.

The CGA intervention concludes once the geriatrician issues their letter and makes the referrals. It does not extend to ensuring a participant’s compliance with the recommendations, the GP actioning recommended medication changes, or a participant’s attendance at any referred allied health interventions.

### Criteria for discontinuing or modifying allocated interventions {11b}

The CGA will only be discontinued at the request of the participant.

### Strategies to improve adherence to interventions {11c}

Completing CGA is routine practice for geriatricians. For the purposes of this trial, and to facilitate standardisation, all geriatricians delivering the consultation will be familiarised with recent consensus guidelines which clearly define CGA [23, 37]. They will also be provided with a template which they can use for their notes if they wish. This template is provided elsewhere (see Supplementary file 3).

### Relevant concomitant care permitted or prohibited during the trial {11d}

It is expected that only a very small percentage of participants in the control group will receive CGA (2–5%) as part of their usual care. This will be recorded in the study database, to allow estimation of contamination. All other aspects of care provided will follow standard local practice for individuals with CKD being managed by a nephrologist.

### Provisions for post-trial care {30}

There are no provisions for post-trial care given the anticipated low risk of harm from a participant’s involvement in this trial.

### Outcomes {12}

Table 2 outlines the outcome measure and timepoints corresponding to each of the trial’s objectives.

**Table 2:** Mapping of outcomes to objectives

Objective	Outcome measure	Timepoint
**Primary objective**		
To determine whether CGA in frail older people with stages 3–5/5D CKD improves attainment of patients’ own goals of care at 3 months	GAS	Baseline and 3 months
**Secondary objectives**		
To determine whether CGA improves attainments of patients’ own goals of care over time	GAS	Baseline, 3, 6 and 12 months
To determine whether CGA improves patient’s quality of life over time	EQ-5D-5L	Baseline, 3, 6 and 12 months
To determine whether CGA favourably affects the trajectory of frailty status over time	FI	Baseline, 3, 6 and 12 months
To determine whether CGA reduces mortality during the 12-month follow-up	All-cause mortality (time to event, proportion) Cause-specific mortality (time to event and proportion)	Continuous
To determine whether CGA reduces the number of hospital admissions during the 12-month follow-up	Hospital admissions (number and reason)	Continuous
To determine whether CGA reduces the hospital length of stay number during the 12-month follow-up	Hospital length of stay (days)	Continuous
To determine whether CGA reduces admissions to RACF at 12 months follow-up	Time to RACF admission	Continuous
To determine whether CGA is cost-effective within the trial context	Health care use analysis	Continuous
**Exploratory objectives**		
Patient recall of health service utilisation	Comparison of data gained from data linkage and the patient-completed Health Care Diary	Continuous
Process evaluation	Qualitative	After completion of the 12-month follow-up
**Safety objective**		
To determine whether CGA is safe when administered to frail older patients with stages 3–5/5D CKD	Any associated hospital admissions or mortality	Continuous

### Participant timeline {13}

**Fig. 1:**
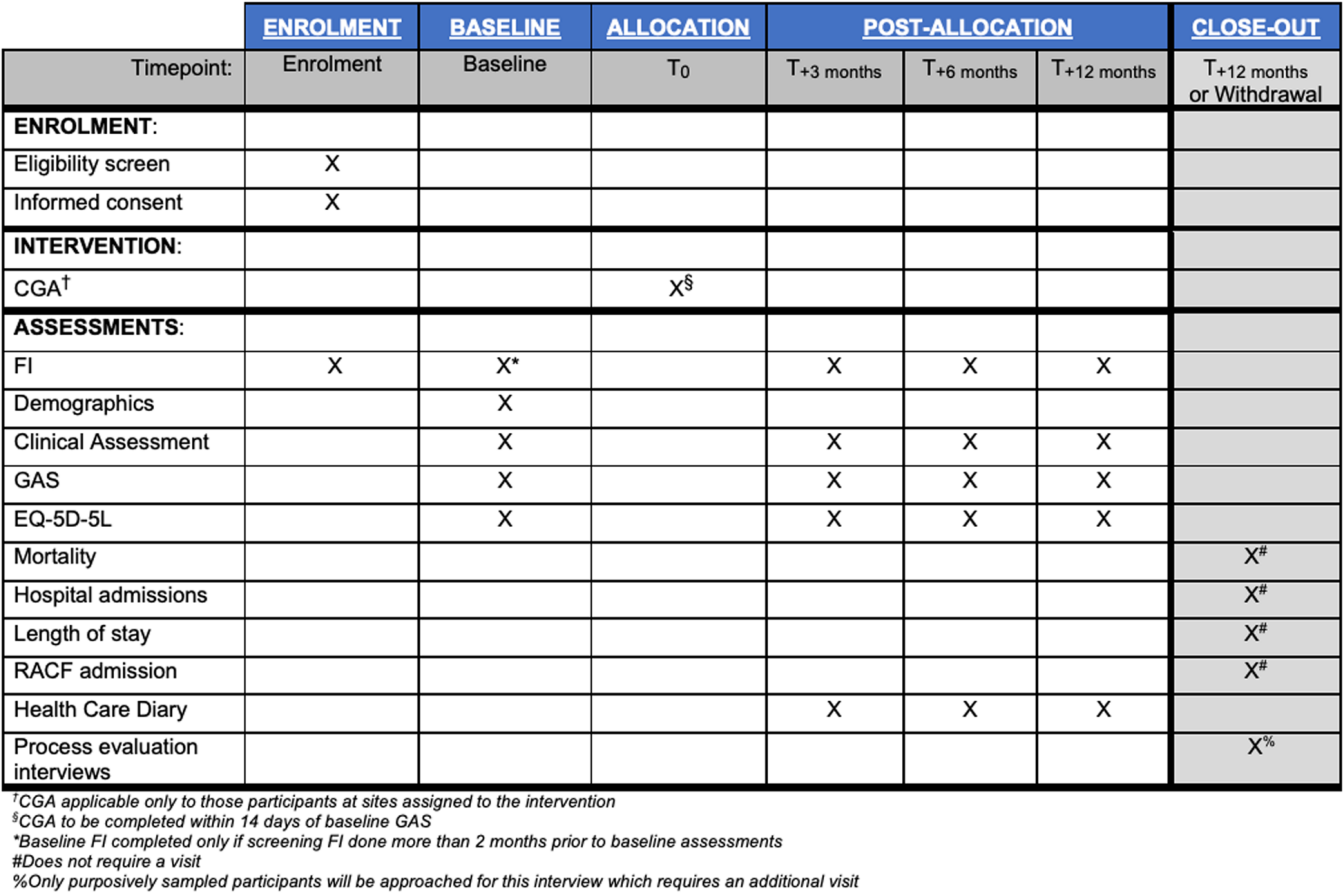
SPIRIT Figure [1, 2] showing the study schedule for enrolment, intervention and assessments

All participants will be followed up by their respective hospitals for a total of 12 months. A SPIRIT [1, 2] diagram for the schedule of enrolment, intervention and assessments is included as Fig. 1. There will be a total of six interactions required for participants at an intervention site and five at control sites. There will be no trial-specific laboratory or radiological investigations requiring visits. Depending on the individual, a geriatrician may request further investigations as part of their CGA and referrals to other specialty services may occur. Upon completion of the trial’s 12-month follow-up, a small group of ~ 30 purposively sampled patients will be approached to participate in interviews for the qualitative data collection required for process evaluation. This interview would necessitate an additional visit.

### Sample size {14}

Based on data from community-dwelling frail older adults, it is estimated that CGA will improve the GAS score by at least 10 points from an average score of 36 at baseline (which presents a change in one standard deviation, equivalent to a medium effect size) [25].

It is estimated that a cluster randomised trial with an average cluster size of 20 participants (coefficient of variation of sizes = 1) would require 16 hospital sites (clusters) with 8 sites in the intervention and control arm, respectively, to achieve 90% power to detect a mean difference in GAS score of 10 units at 3 months follow-up. This power calculation, giving a sample size of 320, assumes a common standard deviation of 10 units and an intra-cluster correlation coefficient of 0.3. To achieve sufficient power for the analysis of the secondary outcome of GAS at 12 months, an allowance for a 33% loss to follow-up over the 12 months was included, requiring baseline GAS observations from 478 participants. The 16 sites will be expected to each have a minimum of 55 patients who meet the eligibility criteria. Based on previous studies, it is estimated that approximately 10% of eligible patients will not consent to participate [26, 29] and a further 35% will not meet the frailty criteria [38] leaving a recruitment target of 500 (approximately 30 at each site). Assumptions in the sample size calculation will be re-estimated on trial data at the mid-point of the planned recruitment period.

### Recruitment {15}

Participants will be recruited from nephrology outpatient departments across the study sites, with the principal investigator, or delegate, identifying potentially eligible individuals from their respective hospital databases. A study invitation letter and information summarising the study may be used by site research staff to inform potential participants that, during an upcoming clinic appointment, they will be approached by their nephrologist, research nurse or delegate about participating in the trial. The letter will include contact information for the research nurse or delegate, should the individual wish to make enquiries about participating.

During clinic appointments, the site’s principal investigator, or delegate will approach prospective trial participants to introduce the trial, describe the study, and answer any questions. This may occur face-to-face or over the phone, depending on the delivery mode of the clinic.

Strategies to achieve adequate participant enrolment include providing clear information in a user-friendly format that outlines the aims of the trial, the individual’s required commitment, and how it is anticipated the research will help people such as them in the future. This will be underpinned by the Consumer Advisory Board reviewing trial participant materials. Other strategies include clear information on who the local contact is for the trial at each site and the provision of a timely response to any questions from prospective participants. Culturally and linguistically diverse patients are accommodated by an interpreter service being available for recruitment and all study assessments.

## Assignment of interventions: allocation

### Sequence generation {16a}

The participating clusters will be randomised to the intervention or control group with an allocation ratio of 1:1, using a block randomisation schedule generated by an independent unblinded statistician.

### Concealment mechanism {16b}

The allocation list will be maintained on a secure server in a folder accessible only to the independent statistician and the block size will be concealed from all other personnel involved in the study.

### Implementation {16c}

All participants at a cluster randomised to an intervention group will receive the intervention, CGA. Participants at a cluster randomised to a control group will receive usual care.

## Assignment of interventions: blinding

### Who will be blinded {17a}

Due to the nature of the intervention complete blinding is not feasible. Therefore, clinicians and participants will not be blinded to the treatment allocation. However, researchers assessing the outcome, responsible for the analysis and reporting of the study (including the lead statistician, lead data manager and trial steering committee members) will be blinded to the allocation of the two intervention arms.

### Procedure for unblinding if needed {17b}

The Data and Safety Monitoring Board and the independent statistician will make recommendations to the Trial Steering Committee, as required, should safety monitoring warrant unblinding.

## Data collection and management

### Plan for assessment and collection of outcomes {18a}

The research nurses or delegate at each study site will be primarily responsible for data collection and data entry, as per the timings overviewed in Fig. 1. They will collect baseline and clinical assessments by meeting with each study participant and reviewing their medical record. They will also administer the GAS, EQ-5D-5L, and FI tools. Data will be directly inputted into Research Electronic Data Capture (REDCap), a secure, web-based software platform hosted at The University of Queensland [39]. Training on data collection processes and the use of REDCap will be provided by the coordinating centre at site initiation.

Given GAS is the primary outcome measure and concerns exist regarding implementation due to the risk of poorly written goals and scales [40–42], a comprehensive training package was developed. This training was designed to ensure the consistent application of GAS by enabling research nurses and delegates to administer it confidently and competently. Supplementary file 4 provides an overview of the training that was provided, which included simulated cases and direct one-on-one feedback.

#### Goal attainment scaling

GAS will be used to measure attainment of patient-identified goals as it is suitable in potentially heterogenous patient groups with differing goals [43, 44], and has been found to be a valid [43, 45, 46], reliable [45, 46], and responsive [25, 47, 48] outcome measure. It has been advocated as a sensitive measure of change in trials of complex interventions for frail older people [25, 49, 50]. As a method of setting measurable goals which are meaningful for patients, it is highly congruent with principles of personalised medicine [51].

The basic steps of GAS include: identifying goals; defining the current (baseline) status; identifying potentially better and worse attainment outcomes on a five-point scale, with consideration of patient and environmental factors such as their current status; weighting the goals; and, at follow-up, scoring the achieved outcome against the stated possible attainment levels [52].

The extent to which goals are achieved is calculated by the formula [53]:

$$\mathrm{GAS score}=50+ \frac{\left[10 \sum \left({w}_{i}\times {x}_{i}\right)\right]}{\sqrt{\left[0.7 \sum {w}_{i}^{2}+0.3 {\left(\sum { w}_{i}\right)}^{2}\right]}}$$

where ${w}_{i}$ = weight assigned to the goal area and ${x}_{i}$ = the attained score for the goal area.

Using this formula, a score of 50 means that all goals have been met. Scores lower than 50 show a deficit in goal attainment, and scores above 50 show better-than-expected outcomes [53]. The overall score is normalised to a mean of 50 and a standard deviation of 10.

#### EQ-5D-5L

Quality of life will be measured by EQ-5D-5L (EuroQol 5 dimensions, 5 levels questionnaire) as it is a frequently used and validated quality of life instrument [54]. The measure also allows comparisons with international data and conversion to a utility score for use in the economic analysis of the intervention.

#### Frailty index

There is a lack of consensus as to how to measure frailty, with a systematic review identifying 20 different frailty instruments used for people with CKD [12]. The FI has been selected for this study as it has been confirmed to be feasible and valid for older people with CKD in the outpatient setting [29]. It also has the degree of granularity necessary to inform decision-making for individuals, which is lacking where dichotomous classifications of frail/not frail are used [29]. Further, it recognises frailty as a multidimensional risk state and, in doing so, it can facilitate quantification of its severity across the health spectrum [29, 55–58].

#### Health care use analysis

Health care use, and their associated costs, will be calculated from the Health Care Diary (see Supplementary file 5) completed by participants and from hospital records when available. The unit cost of care will be derived from appropriate sources for different types of services. For primary care, including specialist visits, the Medicare schedule with estimated out-of-pocket expenses will be applied. For hospital stay, (average) unit cost associated with respective Australian Refined Diagnosis Related Groups (AR-DRG) will be applied. Other costs will be applied at appropriate market wages for the care provided.

#### Patient recall of health service utilisation

Count of service used by health service types collected from the Health Care Diary (participant-completed) will be compared to services recorded and retrieved from data linkage.

#### Process evaluation

At the end of the 12-month follow-up period, qualitative semi-structured interviews will be conducted with ~ 30 purposively sampled patients and their caregivers until data saturation is achieved. Key informant interviews will also be conducted with clinicians (geriatricians, nephrologists, general practitioners, nurses, allied health professionals) involved in the study, with the final number determined by that needed for thematic saturation. All interviews will be recorded and transcribed verbatim.

Additionally, data will be extracted from redacted CGA letters/reports obtained from intervention sites to allow for analysis of the main health and wellbeing issues identified in the CGA and any associated recommendations made by the Geriatrician to address them. An issues register will also be kept by the coordinating centre to capture any observations that can inform the process evaluation.

#### Safety

Data collected for the secondary outcomes related to mortality and associated hospital admissions will be used for monitoring participant safety.

### Plans to promote participant retention and complete follow-up {18b}

Participant retention will be achieved by several strategies. Participant involvement throughout the trial development, activation and conduct, facilitated primarily by obtaining the Consumer Advisory Board’s input, ensures a patient-centred approach is applied to all trial activities and interactions with trial participants. The study staff at each site will be accessible to participants to answer their questions and respond to any concerns. To minimise the burden on a participant’s time, study visits will be scheduled to occur with any pre-existing clinic or other outpatient appointments in place for them when possible. Parking vouchers or travel reimbursements, to a maximum of $60 AUD, will be provided for visits that are study-specific and cannot be done remotely over the phone. Practical guidance and suggestions for participant retention awareness training will occur at the site initiation meetings and is documented in the Operations Manual.

For participants who withdraw from the trial, no further information will be collected from the date of withdrawal. If a participant at an intervention site does not receive the CGA, or a participant at the control site receives a CGA, they will remain in the trial and be followed up until the end of the study and analyses will be conducted using the intention-to-treat principle.

### Data management {19}

Study data will be captured and stored in a REDCap database, which is secure, and password protected. The trial’s data management team will utilise REDCap’s Data Quality module, complemented by R statistical software (R version 4.1.0) [59], for data cleaning and to ensure data completeness, plausibility, and adherence to the protocol. Where potential issues are identified, they will be raised with the study sites to either amend the data or confirm it is correct.

The REDCap database service is managed by the University of Queensland. Original consent forms, paper copies of questionnaires and case report forms will be stored in a secure location accessible only to the local site investigator and the local research nurse at the site where the participant was recruited. These locally stored data will be in identifiable form as they will contain names, dates of birth and other identifiers.

After closure of the trial, investigators will retain all study documentation, including consent documents, ethics committee approvals, and correspondence for a minimum of 15 years or according to local policy before being securely destroyed.

### Confidentiality {27}

Participant records and the data generated by the study will be confidential in line with the recommendations of the NHMRC [60] and Australian privacy legislation [61]. Any information that may identify a participant will be excluded from data presented in the public arena. Data will be stored in a secure, lockable location, and access to electronic data will be protected through a password-protected web interface. The data extracted will be coded using a unique study number. Similarly, data collected on the electronic case report form will be coded using a unique subject number.

### Plans for collection, laboratory evaluation and storage of biological specimens for genetic or molecular analysis in this trial/future use {33}

No additional biological samples outside those collected as part of routine clinical care are being collected from participants.

## Statistical methods

### Statistical methods for primary and secondary outcomes {20a}

Data will be analysed at the individual patient level. The primary outcome, Goal Attainment Scaling (GAS) scores at three months, will be analysed using a linear regression model with the intervention group as a binary covariate and baseline GAS score as a continuous covariate. To allow for the clustering of data within study centres, inference will be based on robust standard errors. Multiple imputation will be used if there is more than 5% missing data on the three-month GAS scores. Subgroup analysis of GAS scores at three months will be undertaken based on baseline characteristics of patients (including age, CKD stage, and frailty status) and study site (including unit size). Subgroup effects will be assessed via treatment-by-subgroup interactions in linear regression models.

Repeatedly measured continuous secondary outcomes (GAS scores at 6 and 12 months, EQ-5D-5L scores, FI) will be analysed using mixed models for repeated measures (MMRM) with a random intercept for the study centres and an unstructured variance–covariance matrix to model the within-patient correlation structure due to the repeated measurements. The models will have fixed effects for the intervention group, categorical time, the intervention-by-time interaction, and baseline measurements of the outcome. The primary results will be the treatment effect estimates at 6 and 12 months and 95% confidence limits.

Other secondary outcomes will be analysed using regression models appropriate for the type of outcome. The effect of CGA on the rate of hospital admission will be tested using a Poisson regression model with a random intercept for study centres and the effect of CGA on time to all-cause mortality and time to hospital admission will be assessed using Cox regression with frailty (random intercepts) for study centres. A detailed statistical analysis plan will be made public before unblinding of the trial statistician and other study personnel at the coordinating centre.

#### Cost-effectiveness evaluation

For the assessment of cost-effectiveness, a cost-utility analysis will be undertaken with the primary analysis from the perspective of the healthcare system. An incremental cost-effectiveness ratio (CER) per quality adjusted life years (QALY) gained in the intervention arm, compared with the usual care arm, will be calculated following the formula:

$$\mathrm{CER}=\frac{{\mathrm{Cost}}_{\mathrm{int}}-{\mathrm{Cost}}_{\mathrm{cont}}}{{\mathrm{QALY}}_{\mathrm{int}}-{\mathrm{QALY}}_{\mathrm{cont}}}$$

where int = intervention group (receiving CGA) and cont = control group (receiving usual care).

Cost of the intervention will be calculated based on additional time over usual care for the nurse and geriatrician to complete their assessments. Costs specific to the research (e.g. research assessments) will not be included. Cost of health care usage in the intervention and control group will be calculated from diaries filled out by participants and from hospital records where available. Unit costs of care will be derived from appropriate sources for the care. For primary care, the Medicare schedule with out of pocket will be applied and average Australian Refined Diagnosis Related Groups (AR-DRG) applied for hospital stays. Other costs will be applied at appropriate wage standards for the care provided.

Utility scores will be derived from the EQ-5D-5L [62] using the Australian algorithm if available (unpublished) or the UK algorithm [63]. Quality-adjusted life years for each group will be calculated using the utility score multiplied by time in the study.

Sensitivity analyses will be conducted for cost variations, intervention durations, as well as EQ-5D-5L score variations. Additional analyses will be conducted should there exist cluster effect and variation across sub-groups (e.g. study location, gender, age, frail levels and other relevant comorbidities). Reporting of the cost-utility analysis will follow the Consolidated Health Economic Evaluation Reporting Standards (CHEERS) guideline [64].

#### Process evaluation

For the process evaluation, descriptive statistical methods will be used to summarise the details of the process of trial performance and CGA delivery, as well as issues noted, including the number and percentages of issues noted in each of the sites, and will also be analysed qualitatively by summarising them in themes, if such themes emerge. The patient and health professional qualitative interviews will be analysed using a thematic framework approach, by extracting themes from the interview transcripts, supported by software (NVivo).

### Interim analyses {21b}

No interim analyses are planned for outcomes. As noted elsewhere, an interim analysis of the sample size is planned.

### Methods for additional analyses (e.g. subgroup analyses) {20b}

Subgroup analysis may be undertaken based on participant (age, CKD stage, frailty) and study site (region, size) characteristics where possible.

### Methods in analysis to handle protocol non-adherence and any statistical methods to handle missing data {20c}

Protocol deviations (e.g. delayed CGA, cross-over) will be investigated in sensitivity analyses. Imputation of randomly missing values will be tested during sensitivity analyses.

### Plans to give access to the full protocol, participant level-data and statistical code {31c}

Access will only be provided after the primary results of the trial and any pre-specified analyses are published. De-identified individual participant data will be made available upon request to a Data Access Committee, a review board set up to assess proposals based on sound science, benefit-risk balancing and research team expertise. Appropriate data will be made available to approved proposals. This process will be in effect for a period of up to 5 years following the publication of the main study results. After 5 years, the data will be available in the Sponsor’s data warehouse but without investigator support other than deposited metadata.

## Oversight and monitoring

### Composition of the coordinating centre and trial steering committee {5d}

The Australasian Kidney Trials Network (AKTN) is the coordinating centre on behalf of the University of Queensland. As the Central Coordinating Centre it is tasked with responsibilities including site set-up and close-out, training, monitoring, data management, statistics, distributions of funds to sites, and supporting the central operations including governance and committees.

The Trial Steering Committee (TSC) is comprised of persons appointed through AKTN procedures, including chief investigators, a statistician and consumers. It is chaired by the Chief Principal Investigator. It is the responsibility of this group to provide leadership to the overall conduct of the trial and ensure trial integrity. In particular, this group will review the progress of the study in achieving the objectives, take appropriate decisions to meet these objectives and make decisions regarding the continuation or modification of the trial given reports from the Data and Safety Monitoring Board. The TSC oversees the trial-related activities of the coordinating centre, AKTN, and reports to the AKTN Scientific Committee.

The Trial Management Committee (TMC) comprises selected chief investigators including the Chair of the TSC, AKTN executive committee member, clinical operations manager, clinical project manager, clinical research associates, statisticians, data managers, and a PhD candidate. The TMC provides support to the TSC in the execution of their responsibilities. Its focus is primarily on operational matters regarding the trial’s management and conduct. It provides regular reports to the TSC with information including site status, recruitment, retention, protocol compliance, data completeness and legal and regulatory issues.

The Consumer Advisory Board comprises nine consumers and a PhD candidate. They provide input on trial design and implementation, and the dissemination of findings, and reports to both the TSC and the TMC. Two consumers are also members of the TSC.

### Composition of the data monitoring committee, its role and reporting structure {21a}

An independent Data and Safety Monitoring Board (DSMB) will monitor safety data, data integrity and trial conduct, and review unblinded data on the incidence and circumstances of safety outcomes. It will be comprised of a nephrologist, a geriatrician and a statistician, all of whom have no financial or scientific conflicts of interest with The GOAL Trial. The DSMB will report to the Chair of the TSC and also the Chair of the AKTN Executive Operations Secretariat.

### Adverse event reporting and harms {22}

No serious adverse events are anticipated to occur as a result of the CGA intervention or assessments (e.g. FI, GAS, EQ-5D-5L) in this trial. Adverse events, such as injurious falls, cardiac events, medication side effects, or death, will be captured by data collected for the secondary outcomes of mortality and hospitalisations. The Data and Safety Monitoring Board will look at trends between the control and intervention groups and action as appropriate.

### Frequency and plans for auditing trial conduct {23}

This study will be monitored by AKTN, as the Central Coordinating Centre, in accordance with International Conference on Harmonisation Good Clinical Practices (ICH GCP), 21CFR Part 312. Monitoring efficiency will be optimised by a system of remote monitoring performed by AKTN. Risk-based monitoring is used for the study. If indicated, and with advance notice, study sites may be visited by a Clinical Monitor. The visits will be an opportunity to provide additional support and training to site staff, ensure the study is conducted according to the protocol, and in line with local regulatory requirements, including Good Clinical Practice. Source documents from which the data are obtained will be made available during the visit to the Clinical Monitor for review. Information garnered through monitoring will be fed back as appropriate to the independent Data and Safety Monitoring Board. The Trial Steering Committee will retain sole decision-making regarding trial continuation and modifications to trial design and procedures while maintaining the confidentiality of the accumulating data.

### Plans for communicating important protocol amendments to relevant parties (e.g. trial participants, ethical committees) {25}

The Trial Management Committee will be responsible for ensuring any protocol amendments are approved by the responsible independent ethics committees and local site governance and then communicated to the principal site investigators and site staff for implementation.

### Dissemination plans {31a}

Trial results will be communicated via publication in scientific journals, through presentations at geriatric medicine, nephrology, and other conferences, and by updates posted on the Australasian Kidney Trials Network website and social media accounts. Participants will have the ability to opt-in to receive information about the trial’s key findings. A summary of the results will be drafted centrally and disseminated by the site’s investigator.

## Discussion

This research directly aligns with the research priorities identified by stakeholders in the care of people living with CKD. The trial includes outcomes identified through the global Standardised Outcomes in Nephrology (SONG) Initiative [65], which involved over 10,000 patients, caregivers, health professionals, and policy makers from more than 100 countries in a consensus process to establish critically important core outcome sets for trials in people living with CKD [66]. Those specific outcomes include improving lifestyle, better treatment to prevent and manage complications and side effects, and access to quality care [67]. Patient-important matters, such as life participation (encompassing the ability to travel), fatigue and mobility [65] are able to be considered by patients as part of the GAS process.

A Consumer Advisory Board has been established to provide input across all stages of the research from designing the study (including strategies for recruitment) through to dissemination (design of plain language reports via print and online media to disseminate the findings to consumers initially via Kidney Health Australia and the broader community) and implementation.

The rollout of the trial’s findings will be supported by workshops and forums with partners (involving ~ 60 participants, caregivers, nephrologists, geriatricians, primary care physicians, and multidisciplinary clinicians, funders, and policy makers) and partner organisations (Australia and New Zealand Society of Nephrology, Caring for Australian and New Zealanders with Kidney Impairment), based upon the SONG process [65] to develop a framework and a pathway for implementation. This will involve plenary sessions and facilitated break-out discussions on the opportunities, barriers, and strategies for implementing the evidence into practice and policy. This will also directly inform the Position Statement on “Management of Frail Older People with Chronic Kidney Disease” planned for endorsement by the Australian and New Zealand Society of Geriatric Medicine.

In recognition of the challenges presented by the COVID-19 pandemic, elements of the trial design and activities have been adjusted to manage the risks to participants and staff and preserve the integrity of the trial. Remote trial activities will be permitted if in-person visits are not feasible. This includes remote delivery of CGA, GAS, and the FI Short Form. All sites are expected to follow local infection control polices related to COVID-19 and manage trial conduct in accordance with applicable local requirements. If a study participant becomes COVID-19 positive during the study, there will be no restrictions on trial participation. The Trial Steering Committee, with the input of both the Trial Management Committee and Consumer Advisory Board as appropriate, may need to make further pragmatic decisions to enable the safe conduct of the trial.

### Trial status

The first trial participant was enrolled on 15 March 2021 and recruitment is expected to be completed by 1 July 2023, with 12-month follow-up of all participants scheduled to be completed by 1 July 2024. The protocol is version 2.5, dated 22 March 2023.

## Supplementary Information

**Additional file 1.**

**Additional file 2.**

**Additional file 3.**

**Additional file 4.**

**Additional file 5.**

## Data Availability

Data sets will be made available by the Trial Steering Committee to researchers within The GOAL Trial network for analysis of sub-studies and post hoc analyses after the primary manuscript has been accepted for publication. For researchers outside The GOAL Trial, de-identified individual participant data will be made available upon request to a Data Access Committee, a review board set up to assess proposals based on sound science, benefit-risk balancing and research team expertise. Appropriate data will be made available to approved proposals. This process will be in effect for a period of up to 5 years following the publication of the main study results. After 5 years, the data will be available in the Sponsor’s data warehouse but without investigator support other than deposited metadata.

## Abbreviations

AKTN: Australasian Kidney Trials Network
AR-DRG: Australian Refined Diagnosis Related Groups
CAB: Consumer Advisory Board
CGA: Comprehensive geriatric assessment
CKD: Chronic kidney disease
CONSORT: Consolidated Standards of Reporting Trials
COVID-19: Coronavirus disease of 2019
DSMB: Data and Safety Monitoring Board
eGFR: Estimated Glomerular Filtration Rate
FI: Frailty Index
GAS: Goal Attainment Scaling
GP: General Practitioner
HREC: Human Research Ethics Committee
ICER: Incremental cost-effectiveness ratio
ICH: International Conference on Harmonisation
NHMRC: National Health and Medical Research Council
QALY: Quality adjusted life year
RACF: Residential Aged Care Facility
SONG: Standardised Outcomes in Nephrology
SPIRIT: Standard Protocol Items: Recommendations for Interventional Trials
TMC: Trial Management Committee
TSC: Trial Steering Committee
