# Supplementary material for: Study protocol for The GOAL Trial: comprehensive geriatric assessment for frail older people with chronic kidney disease to increase attainment of patient-identified goals—a cluster randomised controlled trial
Source: Trials. 2023 May 30;24:365. doi: 10.1186/s13063-023-07363-4 (PMC10227800; doi:10.1186/s13063-023-07363-4)
Supplement: Supplementary file 1 — Additional file 1. [file 13063_2023_7363_MOESM1_ESM.docx]

**The GOAL Trial: List of study sites**

| Austin Health |
| --- |
| Blacktown Hospital |
| Cairns Hospital |
| Concord Repatriation General Hospital |
| Gold Coast Hospital |
| Liverpool Hospital |
| Logan Hospital |
| Princess Alexandra Hospital |
| Renal Research Gosford |
| Royal Adelaide Hospital |
| Royal Perth Hospital |
| Sir Charles Gairdner Hospital |
| Toowoomba Hospital |
| Townsville Hospital |
| Western Health |
