# Supplementary material for: Study protocol for The GOAL Trial: comprehensive geriatric assessment for frail older people with chronic kidney disease to increase attainment of patient-identified goals—a cluster randomised controlled trial
Source: Trials. 2023 May 30;24:365. doi: 10.1186/s13063-023-07363-4 (PMC10227800; doi:10.1186/s13063-023-07363-4)
Supplement: Supplementary file 2 — Additional file 2. [file 13063_2023_7363_MOESM2_ESM.docx]

[Insert site logo]

**Participant Information Sheet/Consent Form**

*Adult providing own consent*

| **Title:** Comprehensive Geriatric Assessment for Frail Older People with Chronic Kidney Disease to Increase Attainment of Patient-Identified Goals - A Cluster Randomised Controlled Trial | |
| --- | --- |
| **Short Title** | The GOAL Trial |
| **Protocol Number** | 20.01 |
| **Project Sponsor** | The University of Queensland, Brisbane |
| **Coordinating Principal Investigator/ Principal Investigator** | *[Coordinating Principal Investigator/*  *Principal Investigator]* |
| **Associate Investigator(s)**  *(if required by institution)* | *[Associate Investigator(s)]* |
| **Location** *(where CPI/PI will recruit)* | *[Location]* |

**Part 1 What does my participation involve?**

**1 What is the GOAL study?**

Geriatricians (doctors who care for older people) use the Comprehensive Geriatric Assessment tool to understand your biggest medical and personal concerns. This helps them to develop a care plan specifically for you. Whilst this is undertaken often with older patients, it is not routinely done for people with chronic kidney disease.

Researchers are hoping (trying) to find out if a Comprehensive Geriatric Assessment by a geriatrician can help the health and wellbeing of people with chronic kidney disease. This will be measured by looking at the achievement of participants’ own goals. Importantly, this study will target the outcomes that are most meaningful to you.

500 participants will take part across 16 hospitals in Australia. The hospitals taking part in the study are allocated to two groups – an ‘intervention group’ (receiving the Comprehensive Geriatric Assessment) and a ‘control group’ (receiving usual care). To try to make sure the groups are the same, the allocation to the intervention and control group will be made at a hospital-level by chance, a bit like tossing a coin.

**2 What does participation in this study involve?**

If you decide to take part, you will be asked to sign a consent form. You will be in the study for 12 months. Please let us know if you need any help with reading or understanding the information sheet. We can arrange support, including finding an interpreter

The study visits will be done after you consent to being involved, and follow up visits three, six and twelve months later. We will do our best to have these done during hospital visits,) or if necessary, we can arrange a telephone appointment.

At your first visit, we will complete the “frailty check” to find out if you are eligible for the study. This will take approximately 15-20 minutes. Some people become frail as they get older or commence dialysis whereas others remain very robust. We can measure frailty by asking about your physical and psychological health and social wellbeing. The more of physical, psychological or social well-being problems you have, the more likely you are to be frail. This study is focusing on people who do have some problems with their health (those who are frailer) rather than people who have very few or no problems (those who are more robust).

- ***If you meet the study frailty requirements***, you can start the study either on the same day or schedule an appointment for another day.
- ***If you do not meet the study requirements***, you will not be able to take part in the study. You will continue to receive your usual care.

If you are eligible for the study:

- We will collect some of your personal information, including health conditions and social supports.
- A research nurse will work with you to set goals that you want to achieve in the next 3 to 12 months. You can choose your own goals, and they can be about anything that is important to you and your quality of life. It may be about how well and far you can walk, or something that will help you with your hobbies or your health. You can bring a support person with you when you set your goals.
- We give you a diary card to record visits you have to specialists or allied health staff like physiotherapists and dietitians and ask to complete a survey.
- We will contact your General Practitioner to inform them that you are taking part in the study and to collect some of your health information. If you are intervention group, we will ask them to organise a referral for Comprehensive Geriatric Assessment.

**If you are in the intervention group** you receive a Comprehensive Geriatric Assessment with a geriatrician after you set your goals. This means coming to your hospital to see the geriatrician (or speaking to the geriatrician over a phone/video call) for an hour appointment. You can bring a support person to the appointment, who can give information about you to the geriatrician to help with the assessment. The doctor will collect information on your medical conditions, social life, daily activities and use of medication, then create a care plan for you. They might refer you to different services like physiotherapy or another specialist if it is required for your health and wellbeing. A summary report will be sent to your kidney doctor and your local General Practitioner.

**Interview sub-study:**

Some people who are in the intervention group will be asked to talk to us at the end of the study about your experiences and your goals. The information collected will help us plan how we might be able to use this intervention for other patients with kidney disease. If you consent and are selected to give feedback, we will contact you to organise a time at the end of the study. You can say no to participating later, even if you consented to the interview in the beginning. The feedback interview will take about 1 hour. We will do this at a time and place that is suitable to you, including by phone or video call. You may pass on any question that makes you feel uncomfortable or distressed. At any time, you may notify the study staff that you would like to stop the interview. The interview will be recorded. All of your answers will be kept confidential.

**3 Do I have to take part in this study?**

Taking part in this study is voluntary. If you do not wish to take part, you do not have to. If you decide to take part and later change your mind, you are free to withdraw from the study at any stage. Your decision whether to take part or not take part, or once you take part and then withdraw, will not affect your usual treatment, your relation with those treating you or your relation with [insert site name].

**4 What are the possible benefits of taking part?**

Taking part in this study may or may not make your health better compared to receiving your usual medical care. The information from this study will help doctors learn more about using Comprehensive Geriatric Assessment in frail older people with kidney disease. This information could help other people with kidney disease in the future.

**5 What are the possible risks and difficulties of taking part?**

We believe that the risks associated with the study are minimal. Some of the questions will focus on your emotions and goals of care. If you become distressed, we can help arrange help and refer you to services where you can get support.

**6 What if I withdraw from this study?**

If you decide to withdraw from the study, please inform a member of the study team before you withdraw. We may be able to find ways to make the trial easier for you to continue if you are having difficulties with some activities. If you do withdraw, you will be asked to complete and sign a short ‘Consent Withdrawal’ form. This will be provided to you by the study team.

If you withdraw your consent during the study, the study doctor and relevant study staff will not collect any more personal information from you. Please note that personal information already collected will be kept to make sure that the results of the study can be measured properly, and to fulfil the requirements of the law, will form part of the study results. If you do not want them to do this, you must tell them before you join the study.

**7 Could this study be stopped suddenly?**

We do not anticipate the study will stop early.

**8 What happens when the study ends?**

Once the study is completed and the results are known, a plain English summary of the results will be made available. You can request a copy of this summary from the study staff. We plan to present and publish the results in scientific journals and meetings. When we publish the results, we will make sure you will not be identified from the information we provide.

**9 Will I be reimbursed for taking part in the study?**

We can provide parking voucher or travel reimbursement of maximum $60 for visits that are study specific and cannot be done remotely over phone.

**Part 2 How is the study being conducted?**

**9 What will happen to information about me?**

When you sign the consent form, you agree to the study team collecting, using and accessing your personal information for the study. Any information obtained in connection with this study that can identify you will remain confidential. It will be disclosed only with your permission, or as required by law. Information about you may be obtained from your health records held at this and other health services like physiotherapy etc and/or General Practitioner for the purpose of this study. Your participation may be recorded in your health records.

Information collected about you will be stored as paper copies in a locked cabinet at [insert name of site] and in the computer on a secure online platform. Your personal details will be collected and stored securely. All information collected from this study will be stored for 15 years after the study is finished. After this, the computer and paper records will be destroyed safely. Only the study team will have access to your details and results while they are stored safely.

Your health records and study information will be examined during the study. This is to confirm the study information has been collected correctly. Reviews may be done by relevant authorities, authorised representatives of the Sponsor, The University of Queensland, the institution listed on this form, [insert site name] or as required by law. By signing this consent form, you agree to the release of, or access to, this confidential information to the bodies listed above.

In addition, information from the study may be shared confidentially for yet undecided studies with researchers not from the study. These studies will be checked for sound science, usefulness/danger balance and team knowledge. Any future studies will progress through ethical review process prior to de-identified data being shared with other researchers. No report of study information will be presented in a way that would recognise or identify you, except with your approval.

In agreement with relevant Australian *and/or* *[Name of state/territory]* privacy and other relevant laws, you have the right to request access to your information collected and stored by the study team. You also have the right to request that any information with which you disagree be corrected. Please contact the study team member named at the end of this document if you would like to access your information.

The state government health information systems will be used to obtain information about inpatient hospital admissions, emergency department visits, survival, use of medications, outpatient services and cost for each visit at the hospital. This is called Data Linkage. The data will be collected from 12 months before you enter into the study to one year after you complete the study. The study team will collect personal information about you, including your hospital UR number, name, date of birth, gender and address. The study sponsor (The University of Queensland) will collect, store and send the above personal details in a safe manner. Using these details, the study team will link your study information with state government health information. Once it is linked, all information provided to the study team members will have your personal information removed (including name, date of birth, and address). Processes have been put in place to make sure that the secrecy of your information is maintained, including removal of information that can tell who you are, the use of special study numbers and obeying to strict guidelines about data transfer, storage and access. If you decide to withdraw your consent for data linkage, this information will not be requested from state government. The data obtained from state linkage will not be shared with external researchers for future unspecified study.

**10 Complaints and compensation**

If you suffer any injuries or complications as a result of this study, you should contact the study team as soon as possible and you will be helped with arranging appropriate medical care. If you are eligible for Medicare, you can receive any medical treatment required to treat the injury or complication, free of charge, as a public patient in any Australian public hospital.

**11 Who is organising and funding the study?**

This research is led by Professor Ruth Hubbard from Princess Alexandra Hospital, and The University of Queensland (UQ), Brisbane, QLD is the sponsor for the study. The research is managed by The Australasian Kidney Trials Network, UQ, and funded by a National Health and Medical Research Council grant. [insert site name] will receive a payment from UQ to assist with the costs of undertaking this study. No member of the study team will receive a personal financial benefit from your involvement in this study (other than their ordinary wages).

**12 Who has reviewed the study?**

All research in Australia involving humans is reviewed by an independent group of people called a Human Research Ethics Committee (HREC). The ethical aspects of this research project have been approved by the HREC of Metro South Health.

This project will be carried out according to the *National Statement on Ethical Conduct in Human Research (2018)*. This statement has been developed to protect the interests of people who agree to participate in human research studies.

The study has assembled a special consumer reference group that has looked at study materials to ascertain they are appropriate and acceptable.

**13 Further information and who to contact**

If you want any further information about this study or if you have any medical problems which may be related to your involvement in the study (for example, any side effects), you can contact the principal study doctor on *[phone number]* or any of the following people:

**Clinical contact person**

| Name | *[Name]* | Telephone | *[Phone number]* |
| --- | --- | --- | --- |
| Position | *[Position]* | Email | *[Email address]* |

For matters relating to study at the site at which you are participating, the details of the local site complaints person are:

**Complaints contact person**

| Name | *[Name]* | Telephone | *[Phone number]* |
| --- | --- | --- | --- |
| Position | *[Position]* | Email | *[Email address]* |

If you have any complaints about any aspect of the study, the way it is being conducted or any questions about being a study participant in general, then you may contact:

**The Responsible HREC approving this research** **and HREC Executive Officer details**

| Name | NA | Telephone | 07 3443 8047 |
| --- | --- | --- | --- |
| Position | Metro South Health Ethics Compliance team | Email | MSH- Ethics@health.qld.gov.au |

**Consent Form -** *Adult providing own consent*

| **Title:** Comprehensive Geriatric Assessment for Frail Older People with Chronic Kidney Disease to Increase Attainment of Patient-Identified Goals - A Cluster Randomised Controlled Trial | |
| --- | --- |
| **Short Title** | The GOAL Trial |
| **Protocol Number** | 20.01 |
| **Project Sponsor** | The University of Queensland, Brisbane |
| **Coordinating Principal Investigator/**  **Principal Investigator** | *[Coordinating Principal Investigator/*  *Principal Investigator]* |
| **Associate Investigator(s)**  *(if required by institution)* | *[Associate Investigator(s)]* |
| **Location** *(where CPI/PI will recruit)* | *[Location where the research will be conducted]* |

**Declaration by Participant**

I have read the Participant Information Sheet or someone has read it to me in a language that I understand.

I understand the purposes, procedures and risks of the study described in the project.

I give permission for my doctors, other health professionals, hospitals or laboratories outside this hospital, state/territory health department to release information to *[Name of Institution]* about my disease, treatment and death for the purposes of this study. I understand that such information will remain confidential.

I have had an opportunity to ask questions and I am satisfied with the answers.

I freely agree to take part in this study as described and understand that I am free to withdraw at any time during the study without affecting my future care.

I understand that, if I decide to stop study visits, a member of the research team may request my permission to get access to my medical records for collection of follow-up information for the purposes of research study and analysis.

I understand as part of the interview I may be audiotaped.

I understand that the information collected about me will be used to support other research in the future, and may be shared anonymously with other researchers.

I understand that I will be given a signed copy of this document to keep.

**Please tick the box to indicate:**

|  |  |  |
| --- | --- | --- |
| I agree to linking of my personal information to state data linkage unit for Hospital Admitted and Non-Admitted, Clinical Costing, Emergency Department and Mortality data for the purpose of the study in a manner that output from linkage will be sufficiently de-identified in a manner that removes all direct identifiers from data. | **YES ☐** | **NO ☐** |
| I agree to take part in the process evaluation interview sub-study | **YES ☐** | **NO ☐** |

|  | | | | | | | |
| --- | --- | --- | --- | --- | --- | --- | --- |
|  | Name of Participant  (please print) | |  | |  |  |  |
|  | | | | | | | |
|  | Signature |  | | Date | |  |  |
|  | | | | | | | |
|  | ***If Required*** Name of Witness* (please print) | |  | |  |  |  |
|  | | | | | | | |
|  | Signature |  | | Date | |  |  |
|  | | | | | | | |

* Witness is not to be the investigator, a member of the study team or their delegate. In the event that an interpreter is used, the interpreter may not act as a witness to the consent process. Witness must be 18 years or older.

**Declaration by Researcher^†^**

I have given a verbal explanation of the research project, its procedures and risks and I believe that the participant has understood that explanation.

|  | | | | | | |
| --- | --- | --- | --- | --- | --- | --- |
|  | Name of Researcher^†^  (please print) | |  | | |  |
|  | | | | | |  |
|  | Signature |  | | Date |  |  |
|  | | | | | | |

^†^ An appropriately qualified member of the research team must provide the explanation of, and information concerning, the research project.

Note: All parties signing the consent section must date their own signature.
