# Supplementary material for: Study protocol for The GOAL Trial: comprehensive geriatric assessment for frail older people with chronic kidney disease to increase attainment of patient-identified goals—a cluster randomised controlled trial
Source: Trials. 2023 May 30;24:365. doi: 10.1186/s13063-023-07363-4 (PMC10227800; doi:10.1186/s13063-023-07363-4)
Supplement: Supplementary file 3 — Additional file 3. [file 13063_2023_7363_MOESM3_ESM.docx]

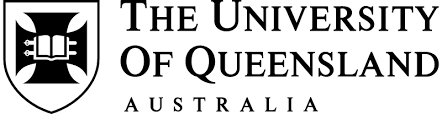

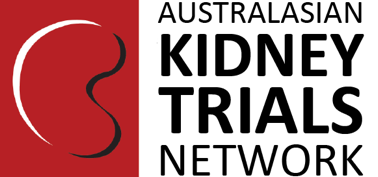

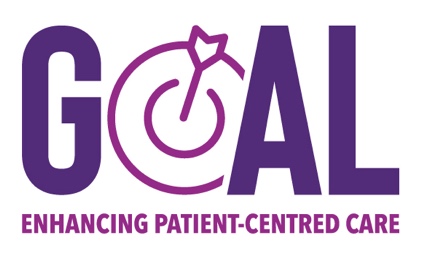


The GOAL Trial: CGA Template

Comprehensive Geriatric Assessment for Frail Older People with Chronic Kidney Disease to Increase Attainment of Patient-Identified Goals - A Cluster Randomised Controlled Trial

Version control

Version: 01.03

Date: 11 November 2020

Explanatory notes

- To be printed on two double-sided A4 pages
- Electronic version to be available for sites using electronic records
- THIS COVER SHEET IS TO BE REMOVED BEFORE PRINTING

Acknowledgements

Adapted from:

- Parker SG, McCue P, Phelps K, McCleod A, Arora S, Nockels K, et al. What is Comprehensive Geriatric Assessment (CGA)? An umbrella review. Age and Ageing. 2018;47(1):149-55.
- Welsh TJ, Gordon AL, Gladman JR. Comprehensive geriatric assessment - a guide for the non-specialist. Int J Clin Pract. 2014;68(3):290-3.

# Comprehensive Geriatric Assessment

Geriatrician: _______________________ Trial ID: _________

GP: _______________________ Date: _________

Nephrologist: _______________________

## Physical Health

### Past Medical History Medications

### Allergies

## Psychological Health

### Cognition

### Mood

## Functioning

### Mobility, balance and falls

### Activities of daily living (incl finances)

### Transport

## Social circumstances

### Social networks (family and informal supports)

### Accommodation

## Future planning

### Advance care planning

### Patient goals and priorities

## Collateral history (family, friend, GP)

## Targeted physical examination

## Key investigations (+/- cognitive testing)

## Assessment and problem list

## Recommendations

### Allied health and other specialist referrals (note whether you are making, or GP is to)

### Medication changes

### Recommendations to the patient

### Other actions (including investigations)
