# Supplementary material for: Study protocol for The GOAL Trial: comprehensive geriatric assessment for frail older people with chronic kidney disease to increase attainment of patient-identified goals—a cluster randomised controlled trial
Source: Trials. 2023 May 30;24:365. doi: 10.1186/s13063-023-07363-4 (PMC10227800; doi:10.1186/s13063-023-07363-4)
Supplement: Supplementary file 4 — Additional file 4. [file 13063_2023_7363_MOESM4_ESM.pdf]

Overview of the Goal Attainment Scaling training package for the GOAL Trial

Comprehensive Geriatric Assessment for Frail Older People with Chronic Kidney Disease to Increase Attainment of Patient-Identified Goals - A Cluster Randomised Controlled Trial

Version control

Version: 01.00

Date: 14 December 2020

Overview

A training package has been developed to enable research nurses, and their delegates, to confidently and consistently administer the Goal Attainment Scaling (GAS) instrument irrespective of their knowledge base and skillset.

The key components of the training package are:

- Formal 'virtual' classroom teaching
  - Scheduled two-hour Zoom-based teaching for groups of around six participants.
  - Includes dedicated practical simulations in small group breakout sessions.
- Self-directed reflection and reading
  - Primarily supported by a training manual (titled "A practical guide to administering Goal Attainment Scaling (GAS) for the GOAL Trial") which:
    - provides a comprehensive step-by-step reference point for the whole GAS process
    - includes a number of tools including a conversation starter and sample populated goals
  - Further resources to support this component include:
    - recording of the 'virtual' classroom teaching session.
    - recorded simulation of an initial goal setting meeting.
- One-on-one simulation and feedback session:
  - Completed 1-2 weeks following the 'virtual' classroom teaching.
  - Conducted by GOAL Trial lead trainer, PhD candidate or delegate.
  - Provides the training participant with the opportunity to practice being a GAS facilitator in a more high-fidelity environment than possible in the breakout classroom teaching.
  - Mechanism for GOAL Trial to ensure proficiency demonstrated by the research nurses, or their delegates. and to provide moderation of consistency between the GAS facilitators.
- Hot review:
  - For the first five goal setting meetings each research nurse or delegate conducts, they will be sent for review to ensure appropriateness of the measures and outcome scaling.
  - Completed by PhD candidate or delegate.

Summary of resources

Training component	Resources	Personnel	Time
Formal 'virtual' classroom teaching	Slides for GAS Classroom teaching for GOAL Trial (PowerPoint slides)<br><br>Simulation scenarios for GAS classroom training (PDF of instructions, and briefing sheets for 'patient' and facilitator')	GAS Lead Trainer<br>(+/- PhD Candidate)	2 and ¼ hours per session<br>- anticipate need for 5 classes<br>- aim for capacity of 6 participants per class
Self-directed reflection and reading	A practical guide to administering Goal Attainment Scaling (GAS) for the GOAL Trial<br>(i.e. the "training manual"), including appendices:<br>- example of populated GAS template<br>- conversation starter guide<br>- example of populated conversation starter guide<br><br>Recording of slide presentation from GAS classroom teaching<br><br>Recording of an example simulation of an initial goal setting meeting	PhD Candidate	3 hours for each nurse
One-on-one simulation and feedback session	Simulation scenario for one-on-one feedback session	GAS Lead Trainer<br>(+/- PhD Candidate)	30 minutes per session<br>(~ 16-25 to be completed)
Hot review		PhD Candidate<br>(+/- GAS Lead Trainer)	15 minutes per review<br>(~ 80-125 to be completed)
