# Supplementary material for: Study protocol for The GOAL Trial: comprehensive geriatric assessment for frail older people with chronic kidney disease to increase attainment of patient-identified goals—a cluster randomised controlled trial
Source: Trials. 2023 May 30;24:365. doi: 10.1186/s13063-023-07363-4 (PMC10227800; doi:10.1186/s13063-023-07363-4)
Supplement: Supplementary file 5 — Additional file 5. [file 13063_2023_7363_MOESM5_ESM.pdf]

ENHANCING PATIENT-CENTRED CARE

Medical Appointments Diary

Participant ID: |___|___|___|___|___| - |___|___|___|

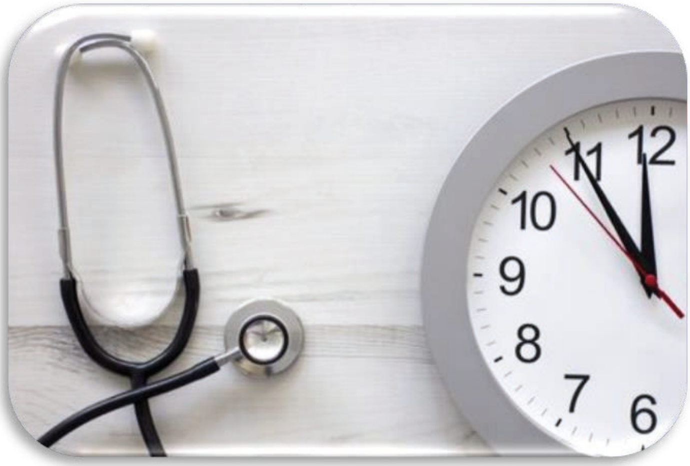

PLEASE USE THIS THROUGHOUT THE GOAL TRIAL

AUSTRALASIAN KIDNEY TRIALS NETWORK
The University of Queensland Australia
Centre for Health Services Research

Version 2.0

Why do I have to complete this diary?

The information will help the research team to study the resources needed to provide consistent and high-quality healthcare in future.

What do I need to do?

- Note your medical or health care appointments or tests
- A new diary will be given to you when you join the GOAL trial and at your 3- and 6-month review appointments
- Complete and return the diary at your next GOAL Trial review appointment

What do I record?

Any medical/health appointments that are day appointments. Some examples are below but you may have others:

- Nephrologist (Kidney)
- Geriatrician (doctor for elderly)
- Surgeon
- Physician
- Psychiatrist
- Ophthalmologist (eye)
- Cardiologist (heart)
- Chiropractor
- Dietitian
- Physiotherapy
- Occupational therapy
- Psychologist
- Respiratory specialist (lung)
- Community nurse
- Massage therapist
- X-rays
- CT scan
- Ultrasound scan
- MRI scan
- PET scan
- Bone scan
- Mammogram

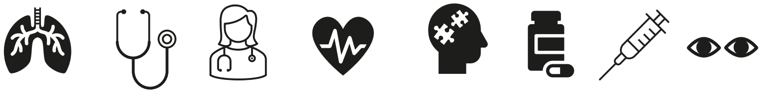

Medical/health appointments

(Excluding overnight hospital stays)

Date	
Appointment/test	

Date	
Appointment/test	

Date	
Appointment/test	

Date	
Appointment/test	

Date	
Appointment/test	

Date	
Appointment/test	

Date	
Appointment/test	

GOAL ENHANCING PATIENT-CENTRED CARE

AUSTRALASIAN KIDNEY TRIALS NETWORK

The University of Queensland Australia | Centre for Health Services Research

Version 2.0

Date	
Appointment/test	

Date	
Appointment/test	

Date	
Appointment/test	

Date	
Appointment/test	

Date	
Appointment/test	

Date	
Appointment/test	

AUSTRALASIAN KIDNEY TRIALS NETWORK

The University of Queensland Australia | Centre for Health Services Research

Date	
Appointment/test	

Date	
Appointment/test	

Date	
Appointment/test	

Date	
Appointment/test	

Date	
Appointment/test	

Date	
Appointment/test	

GOAL
ENHANCING PATIENT-CENTRED CARE

Date	
Appointment/test	

Date	
Appointment/test	

Date	
Appointment/test	

Date	
Appointment/test	

Date	
Appointment/test	

Date	
Appointment/test	

Date	
Appointment/test	

Date	
Appointment/test	

Date	
Appointment/test	

Date	
Appointment/test	

Date	
Appointment/test	

Date	
Appointment/test	

GOAL
ENHANCING PATIENT-CENTRED CARE

Date	
Appointment/test	

Date	
Appointment/test	

Date	
Appointment/test	

Date	
Appointment/test	

Date	
Appointment/test	

Date	
Appointment/test	

Date	
Appointment/test	

Date	
Appointment/test	

Date	
Appointment/test	

Date	
Appointment/test	

Date	
Appointment/test	

Date	
Appointment/test	

GOAL
ENHANCING PATIENT-CENTRED CARE

Thank you for taking part in the GOAL Trial

Contact Us

If you aren't sure what to record or need a new diary, contact

Please use this throughout the GOAL trial
